# Risk factors for antibiotic-resistant bacteria colonisation in children with chronic complex conditions

**DOI:** 10.1038/s41598-022-11295-5

**Published:** 2022-05-04

**Authors:** Martin Agud, Ines de Medrano, Ana Mendez-Echevarria, Talia Sainz, Federico Román, Guillermo Ruiz Carrascoso, Luis Escosa-Garcia, Clara Molina Amores, Francisco José Climent, Aroa Rodríguez, Marta Garcia-Fernandez de Villalta, Cristina Calvo

**Affiliations:** 1 Children’s Medically Complex Diseases Unit, La Paz University Hospital, Paseo de la Castellana, 261, 28046 Madrid, Spain; 2 Paediatric Department, Universidad Autónoma de Madrid, Madrid, Spain; 3 Paediatric Infectious and Tropical Diseases Department, Hospital La Paz Research Institute (IdiPAZ), CIBER Infectious Diseases (ISCIII), La Paz University Hospital, Paseo de la Castellana, 261, 28046 Madrid, Spain; 4 Nosocomial Infections Unit, CNM, Carlos III Health Institute, Majadahonda, Madrid Spain; 5 Department of Clinical Microbiology, La Paz University Hospital, Paseo de la Castellana, 261, 28046 Madrid, Spain

**Keywords:** Microbiology, Microbial ecology

## Abstract

To assess drug-resistant bacterial colonisation rates and associated risk factors in children with complex chronic conditions admitted to a national reference unit in Spain. Cross-sectional study that included all children admitted to our unit from September 2018 to July 2019. Rectal swabs were obtained to determine multidrug-resistant Gram-negative bacilli (MR-GNB) colonisation, and nasal swab to determine *S. aureus* and methicillin-resistant *S. aureus* (MRSA) colonisation. Medical records were reviewed. 100 children were included, with a median of four complex chronic conditions. Sixteen percent had *S. aureus* colonisation, including two MRSA. *S. aureus* colonisation was associated with technology-dependent children, while being on antibiotic prophylaxis or having undergone antibiotic therapy in the previous month were protective factors. The prevalence of MR-GNB colonisation was 27%, which was associated with immunosuppressive therapy (aOR 31; 2.02–47]; *p* = 0.01), antibiotic prophylaxis (aOR 4.56; 1.4–14.86; *p* = 0.012), previously treated skin-infections (aOR 2.9; 1.07–8.14; *p* = 0.03), surgery in the previous year (aOR 1.4; 1.06–1.8; *p* = 0.014), and hospital admission in the previous year (aOR 1.79; [1.26–2.56]; *p* = 0.001). The rate of *S. aureus* nasal colonisation in this series was not high despite the presence of chronic conditions, and few cases corresponded to MRSA. Antibiotic prophylaxis, immunosuppressive therapies, history of infections, previous surgeries, and length of admission in the previous year were risk factors for MR-GNB colonisation.

## Introduction

Drug-resistant bacteria are a public health challenge of growing concern, causing significant mortality and morbidity^1–3^ and with an increasing prevalence, both in healthcare settings and the community^1,4^. Colonisation by certain resistant bacteria might increase the risk of infections^5^, especially in patients undergoing invasive procedures, and poses additional challenges in terms of antimicrobial selection, potentially contributing to an increase in drug-resistant bacteria.

Among the first to garner attention, methicillin-resistant *Staphylococcus aureus* (MRSA) infections emerged in the 1960s, mainly in healthcare settings. Over the next decades, the number of community-associated MRSA (CA-MRSA) infections increased and spread worldwide, with significant differences among regions^6^.

*Staphylococcus aureus* colonizes 20–40% of healthy individuals^7,8^; however, data on paediatric cases are scarce. A previous study (COSACO) by our group found a prevalence of 33% for *S. aureus* nasal colonisation in the community among Spanish children, with a low prevalence of MRSA^9^.

However, there are scarce data regarding the prevalence of MRSA colonisation in children with complex chronic conditions (CCC)^10^, who are known to require frequent hospitalisations, numerous visits to healthcare settings, and the use of invasive medical devices^11^. Schlesinger et al. reported a 36.4% prevalence of *S. aureus* nasal colonisation in children in long-term healthcare institutions in Israel, 21% of whom had MRSA, both rates being significantly higher than the rates in healthy children^7^. The reported risk factors for MRSA colonisation in children include underlying health conditions, recent hospitalisation^12,13^, previous surgery, and neurological disease^13^.

These children can also be colonized by multidrug-resistant Gram-negative bacilli (MR-GNB), which produce extended-spectrum beta-lactamases (ESBL), Amp-C beta-lactamases and carbapenemases^2^. Genes encoding for these enzymes are usually located on plasmids, which contributes to their spread in healthcare settings and the community^2,14^. These plasmids frequently harbour multiple co-resistance genes to other antibiotics. As with MRSA, the prevalence of MR-GNB varies between regions^15^. In countries such as Italy and the US, a progressive increase in the incidence of infections and colonisation due to MR-GNB has been reported in children^16,17^. The risk factors for this colonisation include recent antibiotic use, previous hospitalisation, invasive procedures, and intrafamilial transmission^14,18,19^. In children, similar risk factors have been identified^20–23^, together with specific risk factors such as the presence of underlying neurological conditions^22^, prematurity, low birth weight, and vertical transmission from the mother in the case of neonates. Outbreaks in paediatric and neonatal intensive care units have been reported^2,14^.

In this study, we have analysed the colonization rates for both *S. aureus* and MRSA as well as for MR-GNB, in a unique population who is especially vulnerable to infections, such as children with multiple and severe CCC, trying identify the associated risk factors.

## Materials and methods

A cross-sectional study was performed at the national Spanish Referral Unit for Children with CCC at La Paz University Hospital in Madrid, Spain. Nasal and rectal swabs were obtained from patients younger than 20 years between September 2018 and July 2019. The Clinical Research Ethics Committee (PI18/3346) of La Paz University Hospital approved the study. Informed consent in accordance with the Declaration of Helsinki was obtained from the parents or guardians of all children before their inclusion, as well as from those patients aged 12 years or older, if appropriate.

### Study participants

Children with two or more CCCs and those who were technology-dependent for at least 6 months and had one CCC were included^11^. CCC was defined based on the “*Pediatric Medical Complexity Algorithm”* from the Center of Excellence on Quality of Care Measures for Children with Complex Needs (COE4CCN), *Seattle, Washington*^24^*.* This algorithm defines *CCC as*: (a) any physical, mental, or developmental condition that can be expected to last at least a year, using healthcare resources above the level for a healthy child, requiring treatment to control. This can be expected to be episodically or continuously debilitating; (b) a progressive condition that is associated with deteriorating health with a decreased life expectancy in adulthood^24^.

All patients were screened at admission, following the Unit protocol of weekly screening, and they were included consecutively during the study period. No patient declined the inclusion in the study.

Patients with acute infections and intravenous antibiotic therapy at the time of the study were excluded. Clinical and epidemiological data were retrospectively collected for each patient through a review of the medical records (Supplementary File, supplementary Table S1).

### Study samples for studying *S. aureus* colonisation

Nasal swabs to detect *S. aureus* and MRSA colonisation were collected during the first 48 h after hospital admission and analysed following the standardized protocol of the COSACO study^9,25^. Each sample was collected by inserting the swab tip into the two nostrils and rotating it for 5 s on each side, using ESwab™ flocked swabs with Liquid Amies Medium (Copan, Brescia, Italy)^9^. Methicillin resistance was detected by the disk diffusion method with cefoxitin (30 µg), as well as by susceptibility testing and disk diffusion to other antibiotics^25^. Minimum inhibitory concentrations for MRSA strains were studied with E-test^®^ strips (bioMérieux). Antimicrobial susceptibility was interpreted in accordance with the European Committee on Antimicrobial Susceptibility Testing guidelines^26^. All isolates were screened for the presence of the *mecA* and *mec*C genes using primers by polymerase chain reaction (PCR). Strains in which the *mecA* or *mecC* gene was detected were considered MRSA. Eleven *S. aureus* strains were sent for molecular characterisation to the National Centre of Microbiology (Majadahonda, Spain) for further characterisation (*spa* typing and assessment of the following virulence factors by multiplex PCR reactions: *pvl*, *tst*, *eta*, *etb* and *etd*). In addition, MRSA isolates were characterised by pulsed field gel electrophoresis and staphylococcal cassette chromosome *mec* (*SCCmec*) typing.

### Study samples for studying ESBL and/or carbapenemase-producing Enterobacteriaceae colonisation

Rectal swabs were collected by inserting swabs with Citoswab^®^ semisolid Amies Transport Medium (Citotest Labware Manufacturing, Jiangsu, China) through the anal sphincter and rotating for 10 to 30 s. Samples were cultured on MacConkey agar plates containing 4 mg/L of cefotaxime, and strains were subsequently identified using a matrix-assisted laser desorption ionisation-time-of-flight system (Bruker Daltonics, Bremen, Germany). Phenotypic confirmation of ESBLs and carbapenemases was performed with the double-disk synergy method with cefotaxime, ceftazidime, cefotaxime/clavulanate, and ceftazidime/clavulanate and the modified Hodge test using imipenem, meropenem, and ertapenem discs^27^. PCRs with specific primers were used to detect *bla*_*KPC*_, *bla*_*VIM*_, *bla*_*IMP*_, *bla*_*NDM*_, and *bla*_*OXA-48*_ genes^28^.

### Statistical analysis

The results are presented as absolute frequencies and/or percentages; the quantitative data are expressed as either a median and interquartile range or mean and standard deviation, according to the data distribution. Categorical variables were compared using the chi-squared and Fisher’s exact test, and continuous variables were compared with non-parametric tests. A consecutive multivariate analysis was performed to adjust for potential confounders using logistic regression models. The multivariate analysis included statistically significant variables from the univariate analysis and all variables considered clinically relevant. Nasal colonisation by *S. aureus* and colorectal colonisation by MR GNB were considered dependent variables in these models. A two-sided p-value ≤ 0.05 was considered statistically significant. We calculated the odds ratio (OR) and adjusted OR (aOR) and used them as the association measurement, with 95% confidence intervals (CI) to assess their precision. Stata v17.0 was used for the analysis.

### Ethics approval

The Clinical Research Ethics Committee (PI18/3346) of La Paz University Hospital approved the study.

### Consent to participate

Informed consent in accordance with the Declaration of Helsinki was obtained from the parents or guardians of all children before their inclusion, as well as from those patients aged 12 years or older, if appropriate.

## Results

During the study period, 100 patients were screened (median age 2.3 years [IQR 0.8–5.6]; 60% male). The median number of CCCs per patient was four (IQR 3–5), and 80% were dependent on technology, being the most frequently required devices gastrostomy tube (32%), nasogastric tube (32%), home non-invasive ventilation (31%), home oxygen-therapy (31%), tracheostomy tube (17%) and central permanent central venous access (15%). Ninety-one percent of the patients had been hospitalized in a paediatric intensive care unit at least once in their life, 63% at least once in the last year; and 87% had undergone at least one surgery, with a median of 3 surgeries (1–5). The median intensive care stay during their lives was 30 days (IQR 8–96), and the median hospital stay was 77 days (IQR 30–140).

### *Staphylococcus aureus* colonisation and associated risk factors

*Staphylococcus aureus* was isolated in 16 cases (16%). The study participants’ main characteristics according to *S. aureus* colonisation in the nostrils are summarized in Table 1. Among the 84 non-colonized children, 13 have been intranasally decolonized at least once in their lives with mupirocin, and 20 were receiving antibiotic prophylaxis (11 with cotrimoxazole, 7 with azithromycin and 2 with amoxicillin). None of the colonized patients were receiving antibiotic prophylaxis or have been previously decolonized.

**Table 1:** Factors associated with *S. aureus* nasal colonisation in children with chronic conditions.

	*S. aureus* colonisation N = 16	Non-colonised N = 84	p
**Social conditions**			
Age (years)	2.2 [1.6–5.8]	2.4 [0.8–5.6]	0.731
Male sex	13 (81%)	47 (56%)	0.058
Lives in rural area	3 (19%)	24 (29%)	0.42
Mother as main caregiver	12 (75%)	73 (87%)	0.42
School attendance	10 (62%)	37 (44%)	0.18
Born in Spain	16 (100%)	75 (89%)	0.597
Mother born in Spain	15 (93.7%)	59 (71%)	0.562
Father born in Spain	15 (93.7%)	59 (71%)	0.637
No. of household members	4 [3–5]	3 [3–4]	0.139
No. of siblings	2 [1–3]	2 [1–2]	0.183
**Medical complexity and healthcare use**			
No. of chronic conditions	4 [3–5]	4 [2.5–5]	0.1
Technology dependence	16 (100%)	64 (76%)	**0.029**
Previous surgery	16 (100%)	70 (85%)	0.10
No. of surgeries in the previous year	1 [0–1.5]	1 [0–3]	0.416
No. of surgeries	3.5 [2–6.5]	3 [1–5]	0.295
Total no. of hospitalisations	5.5 [3.5–9.5]	5 [2–8]	0.463
Total length of hospitalisations	77.5 [27.55–135.5]	77 [30–143]	0.926
No. of hospitalisations in the last year	21 [2.5–56]	29 [8–74]	0.293
Total no. of PICU admissions	15 (94%)	75 (90%)	0.67
Length of PICU admission (days)	50 [10.5–102.5]	27.5 [8–86]	0.545
**Previous treatment**			
Antibiotic administration at inclusion	2 (12%)	19 (23%)	0.36
Antibiotic exposure, previous month	2 (12%)	41 (49%)	**0.006**
Antibiotic exposure, previous 6 months	9 (64%)	53 (70%)	0.69
Antibiotic prophylaxis	0 (0%)	20 (24%)	**0.029**
Daily chlorhexidine mouthwash	1 (6%)	1 (1%)	0.19
Inhaled corticoid therapy	8 (50%)	35 (42%)	0.54
Inhaled antibiotic therapy	1 (6%)	4 (5%)	0.80
Immunosuppressive drugs at inclusion	0 (0%)	6 (7%)	0.27
**Previous infections and colonisation**			
Previous skin infections	6 (38%)	22 (27%)	0.37
Previous *S. aureus* infections	3 (19%)	24 (29%)	0.4
Previous *S. aureus* colonisation	1 (6%)	19 (23%)	0.13
Previous nasal decolonisation	0 (0%)	13 (16%)	0.085
MR-GNB colonisation	2 (14%)	22 (29%)	0.24
Previous MR-GNB infections	2 (15%)	20 (25%)	0.46
Previous GNB infections	4 (31%)	42 (51%)	0.17

PICU, paediatric intensive care unit; MR-GNB, multidrug-resistant Gram-negative bacilli.

Significant values are given in bold.

In the univariate analysis, *S. aureus* colonisation was less common in the patients who were non-technology dependent, in those receiving antibiotic prophylaxis, and in those who had undergone antibiotic therapy the previous month (Table 1). A history of infections, hospital admissions, and recent surgery were not associated with a higher risk of *S. aureus* colonisation (Table 1). There were no differences when analysing the association between the number or type of CCC and *S. aureus* colonisation. Due to the small number of colonised patients, the OR could not be calculated for certain risk factors with no events in one of the comparison groups. As a result, multivariate models to predict the risk could not be fitted.

Eleven of these 16 strains were sent to the National Centre of Microbiology to assess the virulence factors and *spa* typing (Supplementary File, supplementary Table S2). Two strains were MRSA, both of them *mecA*-positive and SCCmec type IV, with only three different pattern bands in the pulsed field gel electrophoresis, sharing the same spa type (t002). Both strains were isolated in two patients hospitalised in the same ward at the same time, suggesting possible nosocomial transmission.

### MR-GNB colonisation and associated risk factors

MR-GNB rectal colonisation was present in 24 patients (27%). Twenty-seven resistant strains were isolated in these 24 patients: 16 of which corresponded to ESBL-producing strains, 10 to carbapenemase producers, and 1 producing both ESBL and carbapenemase. The Verona integron-encoded metallo-β-lactamase was the most frequent carbapenemase (6/10). *Klebsiella* spp. was the most frequently isolated MR-GNB (Supplementary File, supplementary Table S3).

The factors associated with MR-GNB colonisation are shown in Table 2. In the univariate analysis, several conditions were identified as potential risk factors for MR-GNB colonisation: younger age, previous antibiotic exposure, history of previous skin infections, immunosuppressive therapies at inclusion, surgery in the previous year, and duration of hospital stays in the previous 12 months. Children who attended school or nurseries presented lower MR-GNB colonisation rates than those who did not attend school or nurseries (*p* = 0.022). No association was found with a history of *S. aureus* or MR-GNB colonisation or infection (*p* > 0.05).

**Table 2:** Factors associated with multidrug-resistant Gram-negative bacilli colonisation in children with chronic complex conditions.

	MR-GNB colonisation N = 24	No MR-GNB colonisation N = 65	p
**Social conditions**			
Age (years)	1 [0.44–1.8]	2.4 [1–5.4]	**0.004**
Male sex	13 (54%)	40 (62%)	0.53
Lives in rural area	5 (21%)	19 (29%)	0.43
Mother as main caregiver	21 (88%)	53 (82%)	0.37
School attendance	5 (21%)	31 (48%)	**0.022**
Born in Spain	21 (87.5%)	71 (94%)	0.422
Mother born in Spain	17 (70%)	49 (77%)	0.404
Father born in Spain	17 (71%)	49 (77%)	0.723
No. of household members	4 [3–5]	4 [3–4]	0.169
No. of siblings	2 [1–3]	2 [1–2]	0.139
**Medical complexity and health-care use**			
No. of chronic conditions	4 [3–5]	4 [3–5]	0.553
Technology dependence	21 (88%)	51 (78%)	0.34
Previous surgery	22 (92%)	53 (84%)	0.36
No. of surgeries in the previous year	3 [2–4]	1 [0–2]	**0.0005**
Total no. of surgeries	3.5 [2–7]	2 [1–5]	0.081
Total no. of hospitalisations	5 [1.5–7.5]	5 [2–9.5]	0.498
Total length of hospitalisations (days)	99 [39–171]	74 [33–138]	0.289
No. of admissions in the last year	2 [1–5.5]	2 [1–4]	0.137
Duration of admissions in the last year (days)	77 [22–153]	26 [8–60]	**0.0006**
PICU admission	24 (100%)	58 (91%)	0.12
Duration of PICU admission (days)	62 [21–101]	25 [9–99]	0.166
**Previous treatment**			
Antibiotic administration at inclusion	4 (17%)	16 (25%)	0.43
Antibiotic exposure, previous month	15 (65%)	25 (38%)	**0.027**
Antibiotic exposure, previous 6 months	20 (91%)	40 (66%)	**0.023**
Antibiotic prophylaxis	9 (38%)	10 (15%)	**0.024**
Daily chlorhexidine mouthwash	0 (0%)	1 (2%)	0.55
Inhaled corticoid therapy	9 (38%)	30 (46%)	0.47
Inhaled antibiotic therapy	1 (4%)	4 (6%)	0.72
Immunosuppressive drugs at inclusion	4 (17%)	2 (3%)	**0.023**
**Previous infections and colonisation**			
Previous skin infections	12 (50%)	16 (25%)	**0.025**
Previous MR-GNB infections	7 (30%)	15 (25%)	0.62
Previous GNB infections	10 (43%)	34 (56%)	0.32
Nasal colonisation, *S. aureus*	1 (4%)	13 (21%)	0.15

PICU, paediatric intensive care unit; MR-GNB, multidrug-resistant Gram-negative bacilli.

Significant values are given in bold.

In the age-adjusted multivariate analysis, factors significantly associated with MR-GNB colonisation were previous skin infections were (aOR 2.9; 1.07–8.14; p = 0.03); immunosuppressive therapies, (aOR 31; 2.02–47; p = 0.01); antibiotic prophylaxis (aOR 4.56; 1.4–14.86; p = 0.012); surgery in the previous year (aOR 1.4; 1.06–1.8; p = 0.014); and hospital admission in the previous year (aOR 1.79; [1.26–2.56*];p* = 0.001). Antibiotic exposure in the previous month or previous 6 months was not associated with MR-GNB colonisation (aOR 2.6; 0.95–7.3; *p* = 0.06 and aOR 3.2; 0.6–16; *p* = 0.16). After adjusting for age, school attendance was not a protective factor. The OR and aOR values for these factors are shown in Table 3.

**Table 3:** Univariate and multivariate analysis of risk factors associated with multidrug-resistant Gram-negative bacilli colonisation.

	OR	p	aOR	p
School attendance	0.29 [0.96–0.87]	0.027	0.47 [0.13–1.66]	0.241
Previous antimicrobial exposure, 1 month	3 [1.1–8.09]	0.03	2.6 [0.95–7.3]	0.06
Previous antimicrobial exposure, 6 months	5.25 [1.12–24.65]	0.036	3.2 [0.6–16]	0.16
Antibiotic prophylaxis	3.3 [1.14–9.58]	0.028	4.56 [1.4–14.86	**0.012**
Immunosuppressive therapy	6.3 [1.07–37]	0.042	31 [2.02–47]	**0.01**
Previous skin infection	3 [1.13–7.99]	0.028	2.9 [1.07–8.14]	**0.03**
Surgery in the previous year	3.8 [1.0–14.2]	0.045	3.34 [0.87–12.75]	0.077
No. of surgeries in the previous year	1.46 [1.13–1.9]	0.04	1.4 [1.06–1.8]	**0.014**
Hospital stay in the previous year (months)	1.88 [1.34–2.64]	< 0.001	1.79 [1.26–2.56]	**0.001**

Significant values are given in bold.

## Discussion

In this study that analysed the colonisation by resistant pathogens in a referral unit for children with CCC, the prevalence of *S. aureus* colonisation was lower than expected, taking into account the features of included patients, with practically no cases of MRSA. However, 1 of every 4 participants had MR-GNB colonisation, and several risk factors associated with the healthcare system were identified, including previous admissions, surgeries, immunosuppressive therapies, and antibiotic prophylaxis. This high rate of MR-GNB could be explained due to the peculiarities of the large referral hospital were the study was performed. Identifying the risk factors can help in implementing strategies for preventing colonisation and optimising treatment in this unique population. The novelty of our study is to study colonization rates in a unique population who is especially vulnerable to infections, analysing both MRSA and MDR-GNB colonization.

The prevalence of *S. aureus* colonisation in this series (16%) is below the 33% reported in community-dwelling children in Spain ^9^, although our included patients were young, and the prevalence of colonization is usually increased in older children^7,9^. However, as we have analyzed children with CCCs, a higher rate of carriage status were expected to be found, at least for MRSA strains, compared to children from the community, as other authors have reported higher colonization rates of *S. aureus* and MRSA in chronically institutionalized children^7^. The use of broad-spectrum antibiotics in children with CCC might have eradicated methicillin-sensitive *S. aureus* and CA-MRSA in this population, as these strains lack other multi-resistance genes^29^. In fact, exposure to antibiotics was also identified as a protective factor for MRSA colonisation in the study by Gesualdo et al.^12^. Half of our non-colonized children had taken antibiotics during the previous month, and up to 70% during the previous 6 months. In addition, none of the children from our cohort who had undergone previous decolonisation were found to be colonised. Antibiotic exposure might have contributed not only to the low prevalence of *S. aureus* and MRSA observed in our cohort but also to the lack of association with previous hospitalisation or surgeries observed in this study. In our study, *S. aureus* colonisation was associated with technology dependency. Other authors have reported higher rates of *S. aureus* colonization in tracheostomized children^30^, or in patients with percutaneous gastrostomy^31^ In the same way, tracheostomy, nasoenteric or gastrostomy tube, or the use of intravenous devices have been reported as risk factors for MRSA acquisition^32–35^. Therefore, invasive devices might favor colonization via bacterial biofilm formation.

The MRSA colonisation rates observed (2%) are similar to those reported in our previous community studies (1.4%)^9^, highlighting the fact that MRSA is not currently a relevant issue among children in Spain. A number of authors have also reported that, in children with CCCs, infections are mostly caused by *Enterobacteriaceae* and *Pseudomonas*, while *S. aureus* has a less relevant role^36,37^. However, other authors have reported a worrisome rise in MRSA cases in other European countries, with an increase specifically among children and youths^4,38^. Epidemiological surveillance is therefore essential.

The risk of *S. aureus* transmission appears to be greater after exposure to other carriers within the household or school/nursery^9,39,40^ than after antibiotic consumption^41^, while resistant *Enterobacteriaceae* colonisation is more frequent when individuals have been exposed to antibiotics^41^. In our cohort, the *S. aureus* colonisation rate did not increase in the children with prolonged hospitalisations, while colonisation appears to be more frequently observed in patients attending schools/nurseries, although our small sample size and low rate of *S. aureus* colonisation did not allow us to observe statistical significances.

Interestingly, the only two MRSA strains that appeared to be genetically related were isolated in patients admitted to the same ward during the same week, being the first admitted case of Latin American origin. Both strains shared the *SCCmec IV* cassette chromosome and were t002 spy type, which is usually found in CA-MRSA^29^. We hypothesize that nosocomial transmission of CA-MRSA strains might have occurred^4^. Higher CA-MRSA rates have been observed in patients of foreign origin in Spain and other European countries, including Latin American populations^4,42^.

There has been a worrying increase in MR-GNB infections in hospital settings in the last decade, with associated high rates of resistance to multiple antimicrobials and high morbidity and mortality^37,43,44^. Although several studies have analysed the prevalence of MR-GNB infections in adult populations, there is paucity of data addressing paediatric populations for whom screening of rectal colonisation is not routinely advised^45^. In addition, a significant variability among different regions and countries has been observed^2,3,15,21^. Our sample consisted of children with CCCs requiring frequent contact with healthcare facilities, use of medical devices, multiple surgeries, intensive care admissions, and recent and prolonged hospitalisations, all of which have been reported as risk factors for MR-GNB infection^2,14,18,21^.

Exposure to broad-spectrum antibiotics, especially carbapenems, fluoroquinolones, and cephalosporin, has been associated with infection by resistant *Enterobacteriaceae*^20,23^. Our data are in line with these findings, given that antibiotic prophylaxis decreased the risk of *S. aureus* colonisation but increased the prevalence of MR-GNB colonisation. In children, the relationship between exposure to non-carbapenem antibiotics and resistant infections is less clear^43^. Unfortunately, the prescribed regimens and administration characteristics were not collected among the study variables and could not be analysed, which, in addition to the sample size, might explain the fact that the association between MR-GNB colonisation and previous antibiotic exposure was non-significant (*p* = 0.06). In our series, immunosuppressive therapy was also associated with MR-GNB colonisation. Other authors have also reported a high rate of infections due to these microorganisms in immunocompromised children^37,43,44^.

Our results highlight the importance of MR-GNB surveillance in this population, to guide empiric antibiotic therapy and implement strict infection control measures to avoid the spread of this worrisome healthcare problem. In addition, there is a need for specific guidelines that stablish the recommendations for routine screening among hospitalised children and among those who have several CCCs. Some authors have reported that patients previously colonized by MRSA, carbapenemase-producing or ESBL-producing *Enterobacteriaceae* might be more susceptible to severe infections once colonized^5,46–48^. This colonization was associated, not only with significantly higher frequency of subsequent infections, but also with increased mortality^48^. Therefore, antibiogram-guided antimicrobial stewardship might be interesting in selected high-risk colonized patients in order to improve prognosis in the case of infections. Knowing colonization state of our patients might help to implement antibiotic stewardship policies in terms of antimicrobial prophylaxis or empirical treatments.

The small sample size and low prevalence of *S. aureus* colonisation in our cohort are the main limitations of this study and impaired our ability to stratify the risk and analyse the potential confounders. In addition, the variability in the ages of included patients probably implies diversity also at the level of the microbiota, with potential impact of age differences on bacterial colonization. The study’s retrospective design precluded us from analysing certain risk factors in detail, such as antibiotic exposure (length, dosing, posology, and type of antimicrobial drug). Lastly, the clinical relevance of MR-GNB colonisation in children with CCCs needs to be clarified. Prospective studies are needed to establish the actual risk of future infections by resistant bacteria in this paediatric group.

In summary, the rate of *S. aureus* colonisation was low in this series of children with CCCs and was associated with technology dependency. Only 2% of the patients were colonised by MRSA. However, we found a high prevalence of MR-GNB colonisation, which was associated with medical complexity and related factors (admissions, surgery, immunosuppressive therapy, antibiotic prophylaxis, and previous infections). Surveillance programs are needed to deepen the knowledge of colonisation and infection risk factors, to optimise management, and to guide empirical therapy in this vulnerable population.

## Supplementary Information

Supplementary Tables.

## Data Availability

Relevant data from our study are provided in the manuscript and the supplementary file.

## Abbreviations

aOR: Adjusted odds ratio
CA-MRSA: Community-associated methicillin-resistant *Staphylococcus aureus*
CCC: Complex chronic conditions
CI: Confidence interval
ESBL: Extended-spectrum beta-lactamases
GNB: Gram-negative bacilli
MRSA: Methicillin-resistant *Staphylococcus aureus*
MR-GNB: Multidrug-resistant Gram-negative bacilli
OR: Odds ratio
SCCmec: *Staphylococcal cassette chromosome mec*
