# Supplementary material for: Risk factors for antibiotic-resistant bacteria colonisation in children with chronic complex conditions
Source: Sci Rep. 2022 May 4;12:7223. doi: 10.1038/s41598-022-11295-5 (PMC9068800; doi:10.1038/s41598-022-11295-5)
Supplement: Supplementary file 1 — Supplementary Tables. [file 41598_2022_11295_MOESM1_ESM.docx]

**RISK FACTORS FOR ANTIBIOTIC-RESISTANT BACTERIA COLONISATION IN CHILDREN WITH CHRONIC COMPLEX CONDITIONS**

**AUTHORS**

**Martin Agud MD^#1)^, Ines de Medrano MD^#2)^**, **Ana Mendez-Echevarria MD, PhD*^3)^**, **Talia Sainz MD, PhD^3)^, Federico Román^4)^,**  **Guillermo Ruiz Carrascoso^5)^,** **Luis Escosa-Garcia MD, PhD^3)^**, **Clara Molina Amores MD^1)^, Francisco José Climent MD, PhD^1)^, Aroa Rodríguez MD^1)^, Marta Garcia Fernandez-deVillalta MD, PhD^1)^, Cristina Calvo MD, PhD^3)^.**

# Contributed equally as co-first authors

***CORRESPONDING AUTHOR: Ana Méndez-Echevarría MD, PhD.

1. Children's Medically Complex Diseases Unit. La Paz University Hospital, Paseo de la Castellana, 261, 28046, Madrid, Spain
2. Paediatric Department. Universidad Autónoma de Madrid, Spain.
3. Paediatric Infectious and Tropical Diseases Department. La Paz University Hospital, Paseo de la Castellana, 261, 28046, Madrid, Spain. Hospital La Paz Research Institute (IdiPAZ); CIBER Infectious Diseases (ISCIII)
4. Nosocomial Infections Unit. CNM. Carlos III Health Institute. Majadahonda. Madrid. Spain
5. Department of Clinical Microbiology. La Paz University Hospital, Paseo de la Castellana, 261, 28046, Madrid, Spain.

**SUPPLEMENTARY FILE**

Supplementary table S1: Epidemiological and clinical variables retrospectively collected:

| Age |
| --- |
| Sex |
| Place of residence (rural or urban area) |
| Child’s and parents’ country of origin |
| Number of household members |
| Number of siblings |
| School/nursery attendance |
| Number and type of CCC |
| Technology dependence (number and type of devices) |
| Antibiotic prophylaxis |
| Inhaled antibiotic therapy |
| Daily chlorhexidine mouthwash |
| Antibiotic therapy at inclusion and in previous months |
| Immunosuppressive drugs at inclusion |
| Previous skin infections |
| Previous *S. aureus* infections |
| Previous *S. aureus* colonisation |
| Previous nasal decolonisation |
| Previous GNB infections |
| Hospitalisation in previous 12 months |
| Duration of hospitalisation in previous 12 months |
| Paediatric intensive care unit admission in the previous 12 months |
| Number of surgeries in the previous 12 months. |

CCC, complex chronic condition, GNB, Gram-negative bacilli

Supplementary table S2: Molecular characterisation of S. aureus strains (11/16)

| **mecA** | **mecC** | **SCC mec** | **PVL** | **TSST** | **ETA** | **ETB** | **ETD** | **spa-type** |
| --- | --- | --- | --- | --- | --- | --- | --- | --- |
| Negative | Negative |  | Negative | Negative | Negative | Negative | Negative | t2527 |
| Negative | Negative |  | Negative | Negative | Negative | Negative | Negative | t012 |
| Negative | Negative |  | Negative | Positive | Negative | Negative | Negative | t822 |
| Negative | Negative |  | Negative | Negative | Positive | Negative | Negative | Unknown |
| **Positive** | Negative | Type IV | Negative | Negative | Negative | Negative | Negative | **t002** |
| Negative | Negative |  | Negative | Negative | Negative | Negative | Negative | t230 |
| Negative | Negative |  | Negative | Negative | Negative | Negative | Negative | t189 |
| Negative | Negative |  | Negative | Positive | Negative | Negative | Negative | t11791 |
| Negative | Negative |  | Negative | Negative | Negative | Negative | Negative | t5995 |
| **Positive** | Negative | Type IV | Negative | Negative | Negative | Negative | Negative | **t002** |
| Negative | Negative |  | Negative | Positive | Negative | Negative | Negative | t822 |

Supplementary table S3: Multidrug-resistant Gram-negative bacilli species and resistance mechanism

| Patient | Species | Resistance mechanism |
| --- | --- | --- |
| 1 | *Klebsiella pneumoniae* | ESBL |
| 2 | *Klebsiella oxytoca* | Carbapenemase VIM |
| 3 | *Klebsiella pneumoniae* | Carbapenemase KPC |
| 4 | *Klebsiella pneumoniae* | ESBL |
| 5 | *Klebsiella pneumoniae* | Carbapenemase VIM |
| 6 | *Klebsiella pneumoniae* | ESBL |
| 7 | *Klebsiella pneumoniae* | Carbapenemase OXA-48 |
| 8 | *Citrobacter freundii* | Carbapenemase VIM |
|  | *Klebsiella oxytoca* | Carbapenemase VIM |
| 9 | *Klebsiella pneumoniae* | Carbapenemase VIM |
| 10 | *Klebsiella pneumoniae* | Carbapenemase VIM  ESBL |
| 11 | *Klebsiella pneumoniae* | ESBL |
| 12 | *Klebsiella pneumoniae* | ESBL |
| 13 | *Klebsiella pneumoniae* | ESBL |
| 14 | *Klebsiella pneumoniae* | ESBL |
| 15 | *Escherichia coli* | Carbapenemase NDM |
| 16 | *Klebsiella pneumoniae* | ESBL |
| 17 | *Klebsiella pneumoniae* | Carbapenemase OXA-48 |
| 18 | *Klebsiella pneumoniae* | ESBL |
| 19 | *Klebsiella pneumoniae* | ESBL |
| 20 | *Klebsiella pneumoniae* | ESBL |
| 21 | *Klebsiella oxytoca* | ESBL |
| 22 | *Klebsiella pneumoniae* | ESBL |
|  | *Citrobacter amalonaticus* | ESBL |
| 23 | *Klebsiella pneumoniae* | ESBL |
| 24 | *Pseudomonas aeruginosa* | Carbapenemase VIM |
|  | *Klebsiella pneumoniae* | ESBL |

ESBL, extended spectrum β-lactamase; VIM, Verona integron-encoded metallo-β-lactamase; KPC, K. pneumoniae carbapenemase; NDM, New Delhi metallo-β-lactamase.
